# Standard Polymeric Formula Tube Feeding in Neurologically Impaired Children: A Five-Year Retrospective Study

**DOI:** 10.3390/nu10060684

**Published:** 2018-05-28

**Authors:** Valeria Dipasquale, Maria Ausilia Catena, Sabrina Cardile, Claudio Romano

**Affiliations:** Pediatric Gastroenterology and Cystic Fibrosis Unit, Department of Human Pathology in Adulthood and Childhood “G. Barresi”, University of Messina, 98123 Messina, Italy; dipasquale.valeria@libero.it (V.D.); mariaausiliacatena@hotmail.com (M.A.C.); sabrinacardile@hotmail.it (S.C.)

**Keywords:** enteral feeding, standard polymeric formula, nutritional outcomes, neurological impairment, children

## Abstract

Malnutrition is frequent in neurologically impaired (NI) children. Enteral feeding via gastrostomy tube is increasingly being used to provide adequate nutrition. Our aim was to assess the outcomes of exclusive gastrostomy tube feeding with standard polymeric formula in children with NI, severe oro-motor dysfunction, and malnutrition, and to investigate the role of the underlying NI-associated disease. A five-year retrospective study from January 2013 to November 2017 was conducted. The primary aim was to assess the nutritional outcomes of exclusive gastrostomy tube feeding with standard polymeric formula in malnourished NI children. The secondary aim was to investigate gastrostomy complications and the impact of the underlying NI-associated disease on the nutritional outcomes. We enrolled 110 consecutive children with NI. Of these patients, 34.5% (*N* = 38) were categorized as malnourished and started exclusive enteral feeding with a standard (1.0 kcal/mL) polymeric formula (Nutrini, Nutricia) after percutaneous endoscopic gastrostomy (PEG) placement. Seventy-three percent of patients (*N* = 28) had cerebral palsy (CP); other diagnoses included metabolic (13%, *N* = 5) and genetic (13%, *N* = 5) diseases. Tricep skinfold thickness had significantly improved in all patients at 12-months follow-up, while body weight and body mass index showed significant increases mainly in children with CP. No serious complications occurred. We found that standard polymeric formula via gastrostomy tube represents a safe and efficient nutritional intervention in children with NI and malnutrition.

## 1. Introduction

Children with neurological impairment (NI) have feeding difficulties secondary to oro-motor dysfunctions and gastrointestinal symptoms, commonly resulting in malnutrition and growth failure. The nutritional management of children with NI was clearly outlined in the 2017 European Society of Gastroenterology, Hepatology, and Nutrition (ESPGHAN) consensus statement [1], which provides uniform recommendations on the proper assessment of nutritional status, diagnosis and treatment of major gastrointestinal symptoms and, above all, timing and modalities of nutritional intervention and rehabilitation. Many feeding strategies have been developed, and multiple approaches may be used in children with NI and malnutrition. ESPGHAN guidelines recommend using a gastrostomy as the preferred way to provide intragastric access for long-term tube feeding. Gastrostomy can be created surgically (preferably laparoscopic), radiology assisted, or endoscopic (percutaneous endoscopic gastrostomy, PEG). The PEG technique has been associated with low rates of intervention failure, discomfort, and interference with social activities [2]. In this study, we have evaluated the nutritional efficacy and tolerance of exclusive gastrostomy tube feeding with standard polymeric formula in undernourished NI children.

## 2. Materials and Methods

A retrospective study of consecutive pediatric patients with NI followed in the Pediatric Gastroenterology Unit of the University Hospital of Messina, Italy, was carried out after IRB approval. Data were collected from January 2013 to November 2017. Inclusion criteria were age ≤ 18 years, and progressive or nonprogressive NI, such as any condition related to the central nervous system, made up of brain and spinal cord, with impairment of the individual’s language, cognitive, and motor skills. There were 110 potentially eligible cases. Variables collection including age, sex, underlying NI-associated disease, major clinical symptoms, and nutritional status were determined. Nutritional status was assessed by using standard anthropometrics (weight and body mass index, BMI) and tricep skinfold thickness (SFT). 

### 2.1. Definition

**Figure 1:**
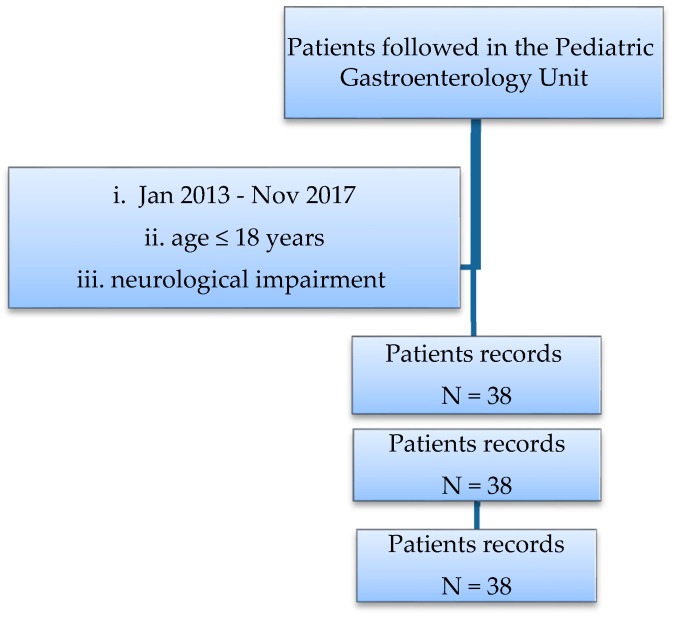
Study flow diagram.

Indications for nutritional rehabilitation included (1) a severe degree of oro-motor dysfunction that compromised nutritional status, as indicated by faltering weight and/or failure to thrive, weight loss, tricep SFT centiles <10th for age and sex, as measured on standard charts; and (2) clinical signs of malnutrition, such as wasting; pale, cold, mottled skin; and poor peripheral circulation. Standard charts used were the World Health Organization growth charts for less than two years of age [3], and the Italian growth charts of Cacciari for older children [4]. Children in whom nutritional rehabilitation was clinically indicated underwent PEG placement by standard pull technique without pH measurements of either acid or non-acid reflux. All children were fed with a standard energy density (1.0 kcal/mL) polymeric formula (Nutrini, Nutricia) with a bolus feeding regimen over the 24-h period. Energy needs were estimated starting from the dietary reference intake (DRI) for basal energy expenditure for typically-developing children [5,6], then individualized, according to mobility, muscle tone, and activity level (based on the Gross Motor Function Classification scale; Palisano and colleagues, 2000). For instance, energy needs for nonambulatory children were approximately 60 to 70% of the estimated average energy requirement for the age for each child. To ascertain how nutritional status had been changing before and after gastrostomy placement, nutritional parameters (body weight, BMI, and tricep SFT) were collected retrospectively from medical records at baseline, namely before gastrostomy placement (visit 1), and at 6 months (visit 2), and 12 months (visit 3) after gastrostomy placement (Figure 1).

### 2.2. Statistical Analysis

Statistical analysis was carried out using univariate analysis or ANOVA test or Wilcoxon rank for continuous data, and the chi-square or Fisher’s exact test for categorical variables. *p* value < 0.05 is the level of statistical significance in all analyses.

## 3. Results

In all 110 consecutive pediatric patients with NI, 34.5% (*N* = 38) met the indications for nutritional rehabilitation. Main demographic and clinical characteristics are reported in Table 1.

The median age of this group of patients was 9.1 years (range: 4 months–18 years); 55.2% were male (*N* = 21). Seventy-three percent of patients (*N* = 28) had cerebral palsy (CP); other diagnoses included metabolic (13%, *N* = 5) and genetic (13%, *N* = 5) diseases. Only 15.78% (*N* = 6) could sit independently and only 7.89% (*N* = 3) could walk unaided. Nearly all of the children (31 out of 38) could not use their hands to feed themselves, and 76.31% (*N* = 29) were unable to grasp any object (equivalent to level V on the Gross Motor Function Classification scale). In addition to motor disabilities, 76.31% (*N* = 29) of children exhibited severe global developmental delay. Oral dysphagia was common (*N* = 32, 84.21%). Potentially dangerous symptoms associated with oro-motor dysfunctions included 57.89% (*N* = 22) of children with cough during meals. At baseline, body weight, BMI, and triceps SFT were ≤5th centile (≤−2.5 *z*-score) in all 38 patients, in comparison with the standards for typically-developing children. Table 2 shows summary statistics for the change in centiles of nutritional parameters between baseline and 12 months after gastrostomy placement. 

Triceps SFT increased substantially over the study period, with a statistically significant improvement (*p* < 0.05) in all 38 patients at 12-months follow-up. Body weight showed significant increases (*p* < 0.05) in 73% (*N* = 28) of children at 12-months follow up, while increase was not significant in the remaining 27% (*N* = 10) children. Similar results were found for BMI, with a statistically significant improvement (*p* < 0.05) in 73% (*N* = 28) of children over the 12-months follow-up. Notably, the group of 28 children who significantly improved in body weight and BMI were those with CP. Body weight increased up to the 10th centile (−1.5 *z*-score) in only three out of five children with metabolic diseases (*p* = 0.2730), and in three out of five children with genetic diseases (*p* = 0.1918). Regarding BMI, only three out of five children with metabolic diseases, and two out of five children with genetic diseases, had an increase in BMI centiles up to the 10th (−1.5 *z*-score), with no significant improvement over the study period (*p* = 0.0828 and *p* = 0.2592, respectively). The complications arising from gastrostomy feeding are shown in Table 3.

No complication occurred during gastrostomy placement. By the second visit (six months after gastrostomy insertion), half (*N* = 14) of the children had the gastrostomy tube changed to a skin-flush button device. No serious complications occurred. None of the children showed a need for fundoplication for worsening of symptoms related to gastroesophageal reflux disease (GERD). None of the children experienced symptoms of feeding intolerance (such as flatulence, distension, and bloating).

## 4. Discussion

### 4.1. Nutritional Outcomes

Enteral feeding via gastrostomy tube is increasingly being used to improve growth and nutritional status of children with NI-associated oral dysphagia and malnutrition. Long-term follow-up studies have shown that PEG is a safe, efficient, and cost-effective feeding technique [7,8,9]. A longitudinal, prospective, multicentre cohort study by Sullivan and colleagues [8,9] enrolled 57 children with CP receiving a gastrostomy with a follow-up of 12 months, and showed a substantial increase in weight gain and SFT over the study period. The children with NI in our study had a severe degree of motor disability. A range of diagnoses were included in our sample (more than three-quarters had CP). After the start of gastrostomy tube feeding with a standard polymeric formula, all children underwent a statistically significant increase in tricep SFT, indicating the deposition of subcutaneous fat in the various sites examined, and 28 of 38 significantly increased in body weight and BMI at 6 and 12 months. As has been observed in other studies [10,11], the overall improving nutritional condition was significantly more represented by SFT than body weight and BMI. Weight measurements do not distinguish between muscle and fat mass percentages. Body mass index has only a moderate correlation with body fat percentage in NI children [10,11]. This is likely due to the abnormal body composition of NI children, who frequently have higher fat percentages and lower lean masses than normally-developing children [12]. A misinterpretation of low BMI or low body weight values may lead to overfeeding, particularly in children who are dependent on gastrostomy tube feeding. As suggested by Samson-Fang and Stevenson [13], tricep SFT measurement is a sensitive, specific predictor of malnutrition, and may represent a more reliable nutritional parameter than standard anthropometrics in NI children. Standard polymeric formula is isocaloric, iso-osmolar (300–350 mOsm/kg), gluten, and lactose free, and has a nutrient composition which is quite similar to that recommended for healthy individuals [14]. In our study, a standard energy density (1.0 kcal/mL) age-appropriate polymeric formula was administered, with a daily intake based on the DRI for basal energy expenditure for sex- and age-matched normally-developing children. Polymeric feed has shown to be effective, well tolerated, and without accentuation of gastrointestinal symptoms such as constipation, gastroesophageal reflux (GER), and retching. Standard polymeric formula is widely recommended by current ESPGHAN guidelines as initial feed of choice, also in the presence of associated gastrointestinal complaints and diseases [1]. 

### 4.2. Gastrostomy Complications

In the 1990s, various research groups debated the impact of gastrostomy tube feeding on the mortality risk of children with NI [15,16]. Strauss and colleagues [16] suggested that the increased mortality associated with tube feeding might be attributable to a differential increase in pulmonary disease secondary to overly vigorous nutritional maintenance and subsequent aspiration after tube placement. Subsequent studies provided evidence supporting gastrostomy as a safe feeding technique. A prospective controlled study enrolling 74 NI children receiving gastrostomy reported that 17% (*N* = 13) experienced at least one major complication, including internal fistula, adhesions, and bleeding, while 82% (*N* = 61) experienced at least one minor complication, including gastrostomy site infection and granuloma [17]. However, a significant difference was found between the type of gastrostomy procedure (PEG versus laparoscopic assisted and associated with fundoplication) and major complications, but not for minor ones. In the prospective study by Sullivan and colleagues [8], they reported an infection rate of 59%, leakage 30%, and granuloma 42%. Only one serious complication (gastric leakage and peritonitis) occurred, in one of the children who had undergone simultaneous laparoscopic gastrostomy and fundoplication. In our study, although minor complications (gastrostomy site infection and granulation tissue) were common, no severe complications after PEG were recorded. None of the patients included in this study had symptoms correlated with abnormal GER after PEG placement. 

### 4.3. The Role of Underlying NI-Associated Diseases

Most children achieved catch-up growth in a relatively short time span as a result of gastrostomy feeding, although there was marked variation; in particular, nutritional intervention appeared to have better nutritional outcomes in children with CP, rather than in those with other diagnoses. The prospective controlled study by Craig and colleagues [17] evaluated health outcomes of gastrostomy for children with NI (*N* = 76) and different underlying diseases—including CP (*N* = 32), genetic (*N* = 25), neurodegenerative (*N* = 11), and not confirmed (*N* = 8) diseases—and found no significant differences in nutritional outcomes between different categories of disabilities. In our study, neither children with genetic disease nor those with metabolic disease experienced significant increases in body weight and BMI over the study period, in comparison to CP children. It is presumable that the quantitative differences between the three patients subgroup, i.e., the exiguity of genetic and metabolic diseases subgroups, could have imparted a significant bias. Furthermore, a favorable influence of the nonprogressive nature of neurological impairment due to CP compared to metabolic diseases with high energy expenditure could be hypothesized, but further studies are warranted. 

We are aware of some limitations. First, this present study is of an observational single-center, retrospective design so recruited cases and clinical management are the confounding variables. Moreover, being a retrospective study, this may be confined only to our clinical findings and management. Consequently, our discussion is not a clinical guideline or consensus for nutritional management of NI children. Second, some missing data are most likely to occur in this present study and also confounders might affect difference of the factors. Third, regarding metabolic and genetic diseases, we did not differentiate between different nosological entities. The strength of this present study is that the indications of nutritional intervention, the timing and modality of enteral support, and the choice of formula are in conformity with current evidence-based guidelines and address a good-quality nutritional strategy for NI children.

## 5. Conclusions

For the vast majority of pediatric patients with NI, standard polymeric formula is sufficient and well tolerated, with the best cost–benefit ratio. It has energy and nutrient content adapted to requirements for age, and can serve as the sole source of nutrition. Percutaneous gastrostomy tube feeding is an effective, long-term nutritional intervention in children with NI and malnutrition, with a good safety profile.

## Figures and Tables

**Table 1:** Demographic and clinical characteristics.

Characteristics	Total (*N* = 38)
Male, *N* (%)	21 (55.2)
Median age (year)	9.1
Underlying disease, *N* (%)	
Cerebral palsy	28 (73)
Metabolic disease	5 (13)
Genetic disease	5 (13)
Major symptoms, *N* (%)	
Oral dysphagia	32 (84.21)
Cough during meals	22 (57.89)
Body weight, *N* (%)	
<−2.5 *z*-score (<5th centile)	23 (60.53)
−1.8 *z*-score (5th centile)	15 (39.47)
−1.5 *z*-score (10th centile)	0
BMI, *N* (%)	
<−2.5 *z*-score (<5th centile)	33 (86.84)
−1.8 *z*-score (5th centile)	5 (13.16)
−1.5 *z*-score (10th centile)	0
Triceps SFT, *N* (%)	
<−2.5 *z*-score (<5th centile)	36 (94.74)
−1.8 *z*-score (5th centile)	2 (5.26)
−1.5 *z*-score (10th centile)	0

BMI, body mass index; SFT, skinfold thickness.

**Table 2:** Nutritional parameters of children at baseline and at 6 and 12 months after gastrostomy.

Disease	Measurement	Baseline (*N* = 38)	Visit 1 (*N* = 38)	Visit 2 (*N* = 38)	*p*
Cerebral Palsy	Body weight, *N* (%)				<0.001
	<−2.5 *z*-score (<5th centile)	18 (64.29)	10 (35.71)	1 (3.57)	
	−1.8 *z*-score (5th centile)	10 (35.71)	10 (35.71)	5 (17.86)	
	−1.5 *z*-score (10th centile)	0	8 (28.57)	22 (78.57)	
	BMI, *N* (%)				<0.001
	<−2.5 *z*-score (<5th centile)	28 (100.00)	20 (71.43)	0	
	−1.8 *z*-score (5th centile)	0	8 (39.57)	2 (7.14)	
	−1.5 *z*-score (10th centile)	0	0	26 (92.86)	
	Triceps SFT, *N* (%)				<0.001
	<−2.5 *z*-score (< 5th centile)	28 (100.00)	10 (35.71)	2 (7.14)	
	−1.8 *z*-score (5th centile)	0	18 (64.29)	10 (35.71)	
	−1.5 *z*-score (10th centile)	0	0	16 (57.14)	
Metabolic Disease	Body weight, *N* (%)				0.273
	<−2.5 *z*-score (<5th centile)	2 (40.00)	1 (20.00)	1 (20.00)	
	−1.8 *z*-score (5th centile)	3 (60.00)	3 (60.00)	1 (20.00)	
	−1.5 *z*-score (10th centile)	0	1 (20.00)	3 (60.00)	
	BMI, *N* (%)				0.0828
	<−2.5 *z*-score (<5th centile)	2 (40.00)	1 (20.00)	1 (20.00)	
	−1.8 *z*-score (5th centile)	3 (60.00)	4 (80.00)	1 (20.00)	
	−1.5 *z*-score (10th centile)	0	0	3 (60.00)	
	Triceps SFT, *N* (%)				0.0126
	<−2.5 *z*-score (<5th centile)	5 (100.00)	2 (40.00)	1 (20.00)	
	−1.8 *z*-score (5th centile)	0	3 (60.00)	1 (20.00)	
	−1.5 *z*-score (10th centile)	0	0	3 (60.00)	
Genetic Disease	Body weight, *N* (%)				0.1918
	<−2.5 *z*-score (<5th centile)	3 (60.00)	1 (20.00)	1 (20.00)	
	−1.8 *z*-score (5th centile)	2 (40.00)	3 (60.00)	1 (20.00)	
	−1.5 *z*-score (10th centile)	0	1 (20.00)	3 (60.00)	
	BMI, *N* (%)				0.2592
	<−2.5 *z*-score (<5th centile)	3 (60.00)	2 (40.00)	1 (20.00)	
	−1.8 *z*-score (5th centile)	2 (40.00)	3 (60.00)	2 (40.00)	
	−1.5 *z*-score (10th centile)	0	0	2 (40.00)	
	Triceps SFT, *N* (%)				0.0477
	<−2.5 *z*-score (<5th centile)	3 (60.00)	1 (20.00)	1 (20.00)	
	−1.8 *z*-score (5th centile)	2 (40.00)	4 (80.00)	1 (20.00)	
	−1.5 *z*-score (10th centile)	0	0	3 (60.00)	

BMI, body mass index; SFT, skinfold thickness.

**Table 3:** Complications of gastrostomy tube feeding within one year of gastrostomy placement.

Complication	Number of Children (%)
Minor site infection	17 (60.71)
Granulation tissue	9 (32.14)
Leakage	5 (17.85)
Tube blockages	3 (10.71)
Tube migration	3 (10.71)
Child pulled tube out	1 (3.57)
